# Explorative study of stimulated saliva proteome in head and neck cancer patients pre- and post-treatment

**DOI:** 10.1016/j.heliyon.2024.e39033

**Published:** 2024-10-09

**Authors:** Ulrica Almhöjd, Amela Fisic, Hülya Cevik-Aras, Lisa Tuomi, Caterina Finizia, Annica Almståhl

**Affiliations:** a Dept of Cariology, Institute of Odontology, Sahlgrenska academy, University of Gothenburg, Sweden; b Section 4- Oral health, Faculty of Odontology, Malmö University, Sweden; c Dept of Oral Pathology and Medicine, Institute of Odontology, Sahlgrenska academy, University of Gothenburg, Sweden; d Specialist Clinic for Orofacial Medicine, Norra Älvsborg County Hospital, Trollhättan, Public Dental Service, Region Västra Götaland, Sweden; e Dept of Otorhinolaryngology, Head and Neck Surgery, Region Västra Götaland, Sahlgrenska University Hospital, Gothenburg, Sweden; f Institute of Neuroscience and Physiology, Speech and Language Pathology Unit, Sahlgrenska Academy, University of Gothenburg, Gothenburg, Sweden; g Dept of Oral Microbiology and Immunology, Institute of Odontology, Sahlgrenska academy, University of Gothenburg, Sweden

**Keywords:** Head and neck cancer, Saliva proteome, Stimulated saliva, Cystatins

## Abstract

**Objectives:**

to compare saliva proteome of patients before treatment of head and neck cancer and six months post-treatment with controls.

**Design:**

Five dentate patients and five age and gender-matched controls were included. The stimulated salivary secretion rate was determined, and saliva was stored at −80 °C. After thawing, 30 mg of each sample and a reference (aliqouts of all samples) was trypsin digested. The digested peptides were analyzed by mass spectrometry. The relative abundances were transformed to log2 and significant differences determined. Relative abundances of mucins were compared with patient's problems with dry mouth, sticky saliva and swallowing. Data are available via ProteomeXchange with identifier PXD047500.

**Results:**

966 proteins with ≥2 unique peptides were found. Compared with controls, 30 proteins were found in significantly lower relative abundances and 65 in higher at pre-treatment and 38 proteins in significantly lower relative abundances and 34 proteins in higher post-treatment. Regarding proteins from the salivary glands, a significantly lower relative abundance of Cystatins was detected pre-treatment and significantly lower relative abundances of Cystatin, Cysteine-rich secretory protein 3, Lactoperoxidase, Prolactin-inducible protein and Proline-rich protein 4 post-treatment. No clear relation between relative abundance of mucins and dry mouth, sticky saliva and problems with swallowing was detected.

**Conclusion:**

Decreases in several salivary gland proteins post cancer treatment might lead to a reduced defense against oral disorders. Knowledge about changes in saliva proteins in connection with oral cancer treatment is important for planning dental care for these patients.

## 1 Background

Almost 600 000 persons/year worldwide get cancer of the head and neck region [1]. Head and neck cancer (HNC) is treated with radiotherapy (RT), surgery, and chemotherapy and often two or three treatment modalities are combined. The oncological treatment can result in early or acute complications such as reduced salivary secretion rate, pain, and fatigue and late complications like trismus, dysphagia, dry mouth, sticky saliva, swallowing difficulties, and difficulties with social eating [[2], [3], [4], [5], [6]]. The oncological treatment reduces quality of life [7]. However, it is not only persons with reduced salivary secretion rates or hyposalivation who report problems with dry mouth, sticky saliva, and swallowing difficulties, but also individuals with normal salivary secretion rates [4,6], indicating that alterations in the saliva composition might contribute to these problems.

A reduction in saliva production usually occurs during the second week of RT [8]. How large the reduction gets is depending on the radiation dose and how much of the salivary gland tissue that is included in the radiation field. Variations in salivary gland size and secretion capacity might also be important for the reduction in salivary secretion rate. Saliva is produced by the major salivary glands (parotid, submandibular, and sublingual glands) and by the 700–1000 minor salivary glands. An unstimulated secretion rate of 0.3–0.4 ml/min and a stimulated secretion rate of 2–3 ml/min are considered normal [9].

Whole saliva contains several hundred to thousands of proteins [[10], [11], [12]]. The largest groups of proteins in saliva are Proline-rich proteins (PRPs; acidic, basic and basic glycosylated proteins), Alpha-amylases, Mucins, Salivary (*S*-type) cystatins, Histatins, Statherin and P-B peptide. Basic PRPs are solely secreted by parotid glands while the submandibular and sublingual glands produce most of the *S*-type cystatins. Acidic PRPs, statherin and P-B peptide are secreted by both the submandibular and sublingual glands [13]. Unstimulated saliva is rich in the mucins MUC5B and MUC7 having important functions for the protection and lubrication of the oral mucosal membranes and affect the feeling of oral dryness. It is also rich in secretory Immunoglobulin A. Stimulated saliva has a high concentration of for example Proline-rich proteins and Amylase [14].

In our previous study, the total protein concentration was increased at 6 months post-treatment compared with pre-treatment, while no significant difference in Immunoglobulin A was detected [15]. At pretreatment, the mucin MUC7 was found in low abundance or not detected at all [15,16], and similar results were found at 6- and 12-months post-treatment [15].

Increased levels of Interleukin-6 (IL-6), IL-8, IL-10, Tumor necrosis factor alpha, chemerin, matrix metalloproteinases 9 have been reported in HNC patients [17]. Increased levels of CYFRA 21-1, lactate dehydrogenase, C reactive protein, carcinomic embryotic antigen, peroxiredoxin-2, zink-alpha-2-glycoproteins, and angiogenic factor proteins have also been found, while both increased levels and levels comparable with controls has been reported for CD44 [17]. The salivary proteome profile has been poorly analyzed both before and after treatment in head and neck cancer patients. To the best of the authors’ knowledge, only one study has shown changes in the salivary proteome during RT and 3–4 months post-treatment [18]. Studies that increase the knowledge about changes in saliva composition especially regarding proteins from the salivary glands may be of importance in the development of more efficient saliva stimulating and saliva substitutes.

The aim of the study was to compare stimulated saliva proteome, especially levels of proteins from the salivary glands, of patients before treatment of head and neck cancer and six months post-treatment with that of age and gender-matched healthy controls with normal salivary secretion rate.

## 2 Methods

The cancer patients were recruited at the multidisciplinary conference at Sahlgrenska University Hospital, a few days before the start of the oncological treatment. Inclusion criteria were that they should be ≥ 18 years old and scheduled for full dose curative treatment of tumors of the head and neck region. Exclusion criteria were age ≥80 years old, poor general health and severe cognitive impairment. Among the saliva samples collected, samples from five dentate patients (3 women and 2 men) where the salivary secretion rate was measured and saliva samples were available from both prior to cancer treatment and six months after completed cancer treatment were included.

Controls matched according to age and gender, with normal salivary secretion rates, not diagnosed with any general diseases, and non-smokers were recruited among employees at the Institute of Odontology, who volunteered to participate and among volunteer patients visiting dentist Anna Adolfsson, Public Dental Care, Stenungsund at their yearly check-ups.

### 2.1 Determination of stimulated salivary secretion rate

In connection with the baseline examination before starting cancer treatment and at six months post-treatment, the stimulated whole salivary secretion rate was determined using paraffin wax. All saliva collections were performed between 10 a.m. and 3 p.m. The vial was stored in a refrigerator at +4 °C for a maximum of 3 h, whereafter it was portioned into Eppendorf vials after cautious vortexing. The Eppendorf vials were stored at −80 °C in the freezer at the Department of Oral Microbiology and Immunology (Sahlgrenska’ biobank, registration number 890.

The saliva samples were transferred to Proteomics, Core Facilities, Sahlgrenska academy. One of the samples from a cancer patient 6 months post-treatment was contaminated with blood and was therefore excluded. After thawing, the samples were centrifuged (21000 g, 20 min, room temperature) to remove debris.

### 2.2 Protein digestion, peptide labeling, and fractionation

In total, 14 samples were used for the proteomic analysis. Pierce™ BCA Protein Assay (Thermo Scientific) was used to determine the total protein concentration with BSA solutions as standards. A reference was made from equal aliquots from all samples (cancer patients pre-treatment and 6 months post-treatment and healthy controls. Samples and the reference (30 μg) were digested with trypsin using the modified filter-aided sample preparation (FASP) method [19]. Briefly, samples were reduced with 100 mM dithiothreitol at 60 °C for 30 min, transferred to Microcon-30kDa Centrifugal Filter Units (catalog no. MRCF0R030, Merck), and washed several times with 8 M urea and once with digestion buffer (0.5 % sodium deoxycholate (SDC) in 50 mM triethylammonium bicarbonate (TEAB) before alkylation with 10 mM methyl methanethiosulfonate in digestion buffer for 30 min. Digestion was performed in digestion buffer by addition of 0.3 μg Pierce MS-grade trypsin (Thermo Fisher Scientific) at 37 °C and incubated overnight. An additional portion of trypsin was added and incubated for another 2 h. Peptides were collected by centrifugation. Digested peptides were labeled using isobaric mass tagging reagents, TMT Pro 16-plex (Thermo Fisher Scientific) according to the manufacturer's instructions.

The labeled samples and the reference were combined into one TMT set, acetonitrile evaporated using vacuum centrifugation, and SDC was removed by acidification with 10 % trifluoroacetic acid and subsequent centrifugation. Further purification was performed using High Protein and Peptide Recovery Detergent Removal Spin Column (Thermo Fisher Scientific) according to the manufacturer's instructions. SDC was removed by acidification with 10 % trifluoroacetic acid (TFA) and subsequent centrifugation. The supernatants were purified using Pierce peptide desalting spin columns (Thermo Fisher Scientific) according to the manufacturer's instructions. The TMT set was pre-fractionated with basic reversed-phase chromatography using a Dionex μltimate 3000 UPLC system (Thermo Fisher Scientific). Peptide separations were performed using a reversed-phase XBridge BEH C18 column (3.5 μm, 3.0 mm × 150 mm, Waters Corporation). The TMT set was collected in fractions, 400 μl/fraction, with a linear gradient from 3 % to 8 % solvent B over 1 min, 8 %–40 % solvent B over 30 min, 40 %–55 % solvent B over 9 min followed by an increase to 100 % B over 5 min and 100 % B for 10 min at a flow of 400 μL/min.

Solvent A was 10 mM ammonium formate buffer at pH 10.00, and solvent B was 90 % acetonitrile and 10 % 10 mM ammonium formate at pH 10.00. The fractions were concentrated into 16 fractions, dried, and reconstituted in 15 μl of 3 % acetonitrile and 0.2 % formic acid.

### 2.3 Nanoflow liquid chromatography/mass spectrometry analysis and database search

Each fraction (5 μl) was analyzed on an Orbitrap Fusion Lumos Tribrid mass spectrometer interfaced with an Easy-nLC 1200 nanoflow liquid chromatography system (Thermo Fisher Scientific). Peptides were trapped on an Acclaim Pepmap 100C18 trap column 100 μm × 2 cm, particle size 5 μm, Thermo Fischer Scientific) and separated on an in-house packed analytical column, particle size 3 μm, Reprosil-Pur C18, Dr. Maisch, 75 μm × 400 mm). Gradients used were; from 5 % to 12 % B over 10 min, 12 %–33 % B over 100 min followed by an increase to 100 % B over 5 min and then 100 % B for 10 min. Flow rate was 300 nL/min. Solvent A was 0.2 % formic acid and solvent B was 80 % acetonitrile in 0.2 % formic acid. Precursor ion mass spectra were acquired at *m*/*z* 200 and 120 000 resolutions, and MS2 analysis was performed in a data-dependent mode where the most intense doubly or multiply charged precursor ions were isolated in the quadrupole with a 0.7 *m*/*z* isolation window and dynamic exclusion within 10 ppm for 45s. The isolated precursors were fragmented by collision induced dissociation (CID) at 30 % collision energy for 3 s (‘top speed’ setting) and detected in the ion trap, followed by multinotch (simultaneous) isolation of the top 10 MS2 fragment ions were fragmented (MS3) by higher-energy collision dissociation (HCD) at or 55 % collision energy and detection in the Orbitrap at 50 000 resolutions.

The data files were merged for identification and relative quantification using Proteome Discoverer version 2.4 (Thermo Fisher Scientific). The search was against Homo Sapiens Swissprot Database (20.376 entries using Mascot 2.5 (Matrix Science) as the search engine with a precursor mass tolerance of 5 ppm and a fragment mass tolerance of 0.5 Da. Tryptic peptides were accepted with zero missed cleavages, variable modifications of methionine oxidation, fixed cysteine alkylation, and TMT-labeled modifications of the N-terminus and lysines. Percolator was used to validate the identified proteins, and the quantified proteins were filtered at 1 % FDR and grouped by sharing the same sequences to minimize redundancy. Only peptides unique for a given protein were considered for quantification of the protein, and ratios were calculated by dividing the samples by the reference sample. The mass spectrometry proteomics data have been deposited to the ProteomeXchange Consortium via PRIDE partner repository with the dataset identifier PXD047500.

### 2.4 Dry mouth, sticky saliva and swallowing

The cancer patients filled in the European Organisation of Research and Treatment of Cancer Quality of Life questionnaire (EORTC-QLQ) head and neck module (H&N35) at pre-treatment and 6 months post treatment. From that questionnaire the answers to the questions about problems with dry mouth, sticky saliva, and swallowing were retrieved. In this questionnaire, the patient is requested to grade his/her problem with for example dry mouth, sticky saliva and swallowing liquids, mashed foods, solid foods, and problems with choking when swallowing. The answering alternatives were not at all (1), a little (2), quite a bit (3), and very much (4).

### 2.5 Statistical analysis

The abundance ratios were transformed to log2 and statistical differences between the healthy persons and the cancer patients at pre-treatment and six months post-treatment as well as for the cancer patients at pre-treatment and at six months post-treatment were calculated using Students' unpaired *t*-test. P-values <0.05 were considered statistically significant. Correlations between patients' problems with dry mouth, sticky saliva, taste and swallowing of liquids, mashed food, solid food, and choking (score 1–4) and the relative abundances of mucins MUC5B and MUC7 at six months post-treatment were analyzed using Spearman's correlation.

### 2.6 Ethical considerations

The study has been approved by the Swedish Ethical Review Board. All subjects were informed about the study and written informed consent was obtained from all participants. All experiments were performed following relevant guidelines and regulations.

## 3 Results

Age, gender, tumor site, treatment, and stimulated salivary secretion rate pre-treatment and 6 months post-treatment are presented in Table 1. The patients had either tonsil cancer or tongue base cancer. Three of the patients were treated with radiotherapy and 2 with chemoradiotherapy. One patient had a low stimulated salivary secretion rate pre-treatment, (0.8 ml/min). At six months post-treatment, 2 patients had very low secretion rates (0.3 and 0.5 ml/min), and 3 a secretion rate within normal limits (≥1 ml/min). In the age and gender-matched controls, the stimulated salivary secretion rate varied from 1.3 to 3.2 ml/min (Table 1).

**Table 1:** Age, gender, tumor site, treatment (RT = radiotherapy, CRT = chemoradiotherapy) and stimulated salivary secretion rate (ml/min) pre-treatment and six months post-treatment for the cancer patients and age, gender, stimulated salivary secretion rate (ml/min) for the healthy matched controls.

Patient	Cancer patients						Healthy controls		
	Age	Gender	Tumor	Treatment	Secretion rate		Age	Gender	Secretion rate
	(years)		Site		Pre-treatment	6 mo post treatment	(years)		
P1	61	F	Tonsil	CRT	0.8	0.3	62	F	2.5
P2	51	F	Tonsil	RT	2.2	2.2	48	F	3.0
P3	76	F	Tonsil	RT	1.8	1.0	72	F	3.0
P4	55	M	Tongue base	RT	2.2	1.2	58	M	1.3
P5	63	M	Tongue base	CRT	1.0	0.5	59	M	3.2

Mean ± SD	61 ± 10				1.6 ± 0.7	1.3 ± 1.2	60 ± 9		2.6 ± 0.8

### 3.1 Proteome

966 proteins with ≥2 unique peptides were found.

### 3.2 Proteins in cancer patients compared with healthy controls

30 of the proteins were found in lower relative abundances in the cancer patients at pre-treatment compared with the control group (13 enzymes, 9 cell-associated, 7 associated with the immune system, 1 salivary gland protein) and 65 proteins in higher relative abundances (41 cell-associated, 16 enzymes, 4 associated with the immune system, 3 other, 1 protease inhibitor) (Table 2).

**Table 2:** Significant changes in relative abundances (%) of proteins in stimulated whole saliva in cancer patients pre-treatment compared with healthy controls.

Accession	Protein name	Cancer	Healthy	p-value
Number		pre-treatment	controls	
Lower in cancer patients pre-treatment				
Q11128	4-galactosyl-N-acetylglucosaminide 3-alpha-L-fucosyltransferase FUT5	1.04 ± 0.27	1.76 ± 0.50	0.020
P49189	4-trimethylaminobutyraldehyde dehydrogenase	0.79 ± 0.22	1.35 ± 0.51	0.043
P08195	4F2 cell-surface antigen heavy chain	1.02 ± 0.32	1.64 ± 0.58	0.048
P40394	All-trans-retinol dehydrogenase [NAD(+)] ADH7	0.79 ± 0.33	1.51 ± 0.52	0.030
P54802	Alpha-N-acetylgalactosaminidase	0.88 ± 0.07	1.25 ± 0.23	0.012
P03933	Antileukoproteinase	0.35 ± 0.17	0.69 ± 0.38	0.044
Q13867	Bleomycin hydrolase	0.96 ± 0.13	1.26 ± 0.12	0.006
O75976	Carboxypeptidase D	0.86 ± 0.32	1.58 ± 0.57	0.048
P13688	Carcinoembryonic antigen-related cell adhesion molecule 1	0.68 ± 0.23	1.04 ± 0.17	0.049
P13987	CD59 glycoprotein	0.68 ± 0.16	0.95 ± 0.20	0.043
P29373	Cellular retinoic acid-binding protein 2	0.77 ± 0.22	1.32 ± 0.53	0.045
P07358	Complement component C8 beta chain	1.08 ± 0.31	1.75 ± 0.20	0.009
P08174	Complement decay-accelerating factor	0.82 ± 0.14	1.40 ± 0.40	0.010
P01037	Cystatin-SN	1.29 ± 0.93	2.54 ± 0.41	0.043
Q02487	Desmocollin-2	0.99 ± 0.27	1.49 ± 0.31	0.018
P32926	Desmoglein-3	0.98 ± 0.32	1.56 ± 0.35	0.028
O94919	Endonuclease domain-containing 1 protein	0.97 ± 0.22	1.61 ± 0.40	0.009
Q12841	Follistatin-related protein 1	0.82 ± 0.36	1.57 ± 0.49	0.031
P09958	Furin	0.93 ± 0.35	2.03 ± 1.00	0.019
P41250	Glycine-tRNA ligase	0.91 ± 0.04	1.02 ± 0.09	0.044
A0A0B4J1Y9	Immunoglobulin heavy variable 3-72	0.86 ± 0.11	1.32 ± 0.41	0.033
Q04695	Keratin, type I cytoskeletal 17	0.40 ± 0.09	0.83 ± 0.41	0.038
O95867	Lymphocyte antigen 6 complex locus protein G6c	0.90 ± 0.32	2.04 ± 0.75	0.012
P40121	Macrophage-capping protein	1.05 ± 0.32	1.45 ± 0.19	0.043
P08582	Melanotransferrin	0.95 ± 0.15	1.51 ± 0.42	0.017
Q86SF2	N-acetylgalactosaminyltransferase 7	0.73 ± 0.22	1.42 ± 0.32	0.013
Q16651	Prostasin	1.01 ± 0.28	1.55 ± 0.33	0.031
Q9HAT2	Sialate O-acetylesterase	0.91 ± 0.11	1.37 ± 0.40	0.041
Q86T26	Transmembrane protease serine 11B	0.71 ± 0.27	1.89 ± 1.41	0.024
Q9UL52	Transmembrane protease serine 11E	0.87 ± 0.35	1.38 ± 0.34	0.049
Higher in cancer patients pre-treatment				
Q04917	14-3-3 protein eta	1.13 ± 0.24	0.82 ± 0.14	0.038
015511	Actin-related protein 2/3 complex subunit 5	1.25 ± 0.30	0.78 ± 0.30	0.040
Q9BPX5	Actin-related protein 2/3 complex subunit 5-like protein	1.11 ± 0.10	0.81 ± 0.14	0.010
P55008	Allograft inflammatory factor 1	1.55 ± 0.61	0.92 ± 0.19	0.028
Q9ULZ3	Apoptosis-associated speck-like protein containing a CARD	1.04 ± 0.15	0.73 ± 0.16	0.021
Q9UBW5	Bridging integrator 2	1.22 ± 0.24	0.66 ± 0.20	0.008
P11586	C-1-tetrahydrofolate synthase, cytoplasmic	1.51 ± 0.35	0.88 ± 0.33	0.026
Q96CX2	Calcium-binding protein 39	1.09 ± 0.14	0.63 ± 0.31	0.041
P23528	Cofilin-1	1.16 ± 0.12	0.81 ± 0.15	0.009
P01024	Complement C3	1.15 ± 0.18	0.78 ± 0.20	0.022
P00751	Complement factor B	1.06 ± 0.20	0.72 ± 0.12	0.010
Q13561	Dynactin subunit 2	1.27 ± 0.23	0.80 ± 0.14	0.003
Q9H449	EH domain-containing protein 1	1.22 ± 0.27	0.72 ± 0.32	0.045
P24534	Elongation factor 1-beta	1.01 ± 0.17	0.65 ± 0.18	0.021
P29692	Elongation factor 1-delta	1.16 ± 0.16	0.64 ± 0.28	0.044
P26641	Elongation factor 1-gamma	1.21 ± 0.23	0.78 ± 0.26	0.035
P14625	Endoplasmin	1.29 ± 0.30	0.69 ± 0.29	0.023
P23588	Eukaryotic translation initiation factor 4B	1.34 ± 0.40	0.73 ± 0.19	0.009
P55060	Exportin-2	1.74 ± 0.40	0.88 ± 0.47	0.035
Q12841	Fructose-bisphosphate aldolase C	1.10 ± 0.13	0.86 ± 0.14	0.019
P09488	Glutathione S-transferase Mu 1	1.46 ± 0.66	0.13 ± 0.02	0.0003
P62993	Growth factor receptor-bound protein 2	2.02 ± 1.09	0.75 ± 0.35	0.024
P00738	Haptoglobin	1.37 ± 0.60	0.64 ± 0.21	0.041
P07900	Heat shock protein HSP 90-alpha	0.98 ± 0.16	0.55 ± 0.23	0.024
P08238	Heat shock protein HSP 90-beta	1.07 ± 0.27	0.53 ± 0.24	0.031
Q32P51	Heterogeneous nuclear ribonucleoprotein A1-like 2	1.34 ± 0.54	0.73 ± 0.21	0.025
O60812	Heterogeneous nuclear ribonucleoprotein C-like 1	1.08 ± 0.22	0.68 ± 0.18	0.017
Q14103	Heterogeneous nuclear ribonucleoprotein D0	1.37 ± 0.34	0.75 ± 0.20	0.006
P31943	Heterogeneous nuclear ribonucleoprotein H	1.29 ± 0.39	0.80 ± 0.19	0.024
P61978	Heterogeneous nuclear ribonucleoprotein K	1.10 ± 0.21	0.78 ± 0.19	0.030
P22626	Heterogeneous nuclear ribonucleoproteins A2/B1	1.57 ± 0.63	0.82 ± 0.42	0.039
P19367	Hexokinase-3	1.34 ± 0.44	0.86 ± 0.21	0.041
P0DOX5	Immunoglobulin gamma-1 heavy chain	1.00 ± 0.25	0.59 ± 0.21	0.022
Q7Z4W1	L-xylulose reductase	1.03 ± 0.22	0.66 ± 0.23	0.035
P26038	Moesin	1.16 ± 0.38	0.69 ± 0.20	0.026
P43490	Nicotinamide phosphoribosyltransferase	1.33 ± 0.62	0.64 ± 0.17	0.035
Q6XQN6	Nicotinate phosphoribosyltransferase	1.30 ± 0.38	0.78 ± 0.22	0.034
P19338	Nucleolin	1.25 ± 0.34	0.68 ± 0.31	0.030
Q6P4A8	Phospholipase B-like 1	1.09 ± 0.40	0.48 ± 0.21	0.018
Q15365	Poly(rC)-binding protein 1	1.14 ± 0.18	0.82 ± 0.18	0.034
Q06323	Proteasome activator complex subunit 1	1.09 ± 0.16	0.81 ± 0.19	0.048
P30101	Protein disulfide-isomerase A3	1.13 ± 0.32	0.77 ± 0.12	0.048
Q15084	Protein disulfide-isomerase A6	1.03 ± 0.16	0.74 ± 0.15	0.025
Q15435	Protein phosphatase 1 regulatory subunit 7	1.10 ± 0.13	0.71 ± 0.26	0.029
O75688	Protein phosphatase 1B	1.22 ± 0.21	0.85 ± 0.24	0.035
Q12913	Receptor-type tyrosine-protein phosphatase eta	1.12 ± 0.29	0.74. 0.24	0.049
P52565	Rho GDP-dissociation inhibitor 1	1.08 ± 0.20	0.75 ± 0.21	0.043
P52566	Rho GDP-dissociation inhibitor 2	1.48 ± 0.51	0.88 ± 0.09	0.025
Q07960	Rho GTPase-activating protein 1	1.48 ± 0.45	0.90 ± 0.31	0.036
P98171	Rho GTPase-activating protein 4	1.45 ± 0.64	0.84 ± 0.23	0.038
O95747	Serine/threonine-protein kinase OSR1	1.58 ± 0.25	1.23 ± 0.16	0.034
Q08209	Serine/threonine-protein phosphatase 2B catalytic subunit alpha isoform	1.27 ± 0.29	0.85 ± 0.18	0.020
P48595	Serpin B10	1.40 ± 0.31	0.86 ± 0.18	0.010
P35542	Serum amyloid A-4 protein	1.18 ± 0.43	0.50 ± 0.18	0.027
P16949	Stathmin	1.57 ± 0.59	0.81 ± 0.24	0.021
P31948	Stress-induced-phosphoprotein 1	1.29 ± 0.16	0.98 ± 0.24	0.015
O15400	Syntaxin-7	1.31 ± 0.43	0.81 ± 0.22	0.031
P50991	T-complex protein 1 subunit delta	1.19 ± 0.22	0.82 ± 0.23	0.036
P05543	Thyroxine-binding globulin	1.28 ± 0.27	0.72 ± 0.22	0.019
O75347	Tubulin-specific chaperone A	1.31 ± 0.22	0.92 ± 0.22	0.021
O95777	U6 snRNA-associated Sm-like protein LSm8	1.23 ± 0.29	0.87 ± 0.22	0.039
P41226	Ubiquitin-like modifier-activating enzyme 7	1.66 ± 0.62	0.90 ± 0.27	0.025
P38606	V-type proton ATPase catalytic subunit A	1.09 ± 0.17	0.80 ± 0.21	0.045
P08670	Vimentin	1.23 ± 0.33	0.80 ± 0.25	0.031
P13010	X-ray repair cross-complementing protein 5	1.23 ± 0.36	0.67 ± 0.27	0.042

**Fig. 1:**
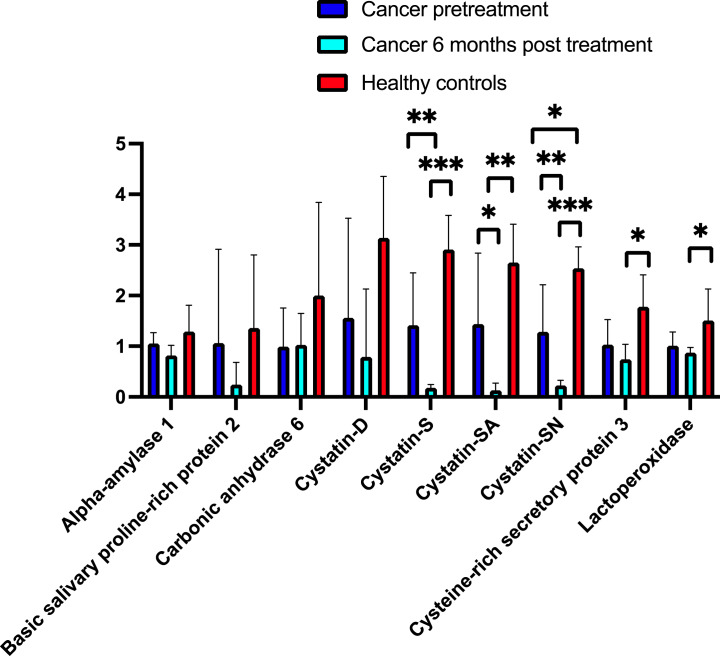

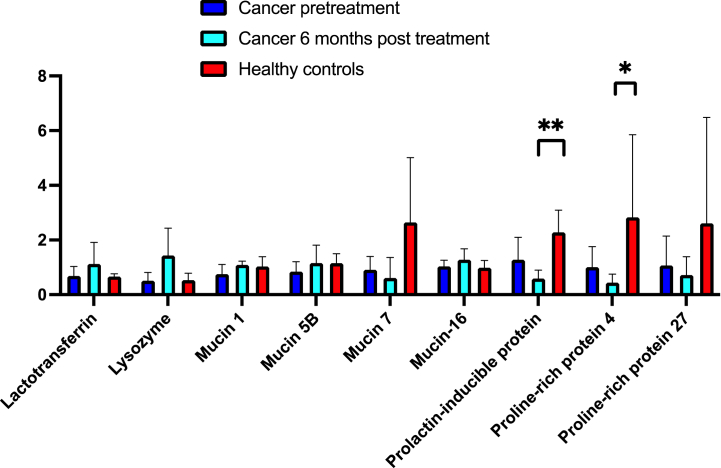
Fig. 1a Relative abundance (%) of common saliva proteins in stimulated saliva in cancer patients pre-treatment (n = 5), six months post-treatment (n = 4) and in healthy controls (n = 5). Fig. 1b Relative abundance (%) of common saliva proteins in stimulated saliva in cancer patients pre-treatment (n = 5), six months post-treatment (n = 4) and in healthy controls (n = 5).

In Fig. 1a and b, relative abundances of 16 common saliva proteins in the cancer patients pre-treatment and 6 months post-treatment, and in the healthy controls are shown. The cancer patients had a lower relative abundance of Cystatin SN compared with the healthy controls (Fig. 1a). Tendencies to lower relative abundances of Cystatin S (p = 0.05), Cystein-rich secretory protein 3 (p = 0.06), and Mucin MUC7 (p = 0.07) in the cancer patients were also detected.

At six months post-treatment, 38 proteins were found in lower relative abundances in the cancer patients compared with the control group (14 cell-associated, 9 enzymes, 7 associated with the immune system, 7 salivary gland proteins, 1 protease inhibitor) and 34 proteins in higher relative abundances (19 cell-associated, 11 enzymes, 3 associated with the immune system, 1 protease inhibitor) (Table 3).

**Table 3:** Proteins showing statistically significant differences in relative abundances (%) between cancer patients with 6 months post-treatment compared with healthy controls.

Accession	Protein name	Cancer 6 months	Healthy	p-value
Number		post-treatment	controls	
Lower in cancer patients				
O43598	2′-deoxynucleoside 5′-phosphate N-hydrolase 1	0.77 ± 0.26	1.29 ± 0.21	0.027
Q11128	4-galactosyl-N-acetylglucosaminide 3-alpha-L-fucosyltransferase FUT5	0.77 ± 0.40	1.76 ± 0.50	0.037
Q9BRK5	45 kDa calcium-binding protein	0.75 ± 0.27	2.02 ± 1.31	0.044
P08195	4F2 cell-surface antigen heavy chain	0.70 ± 0.09	1.64 ± 0.58	0.003
Q16706	Alpha-mannosidase 2	1.14 ± 0.16	1.42 ± 0.12	0.037
O43505	Beta-1,4-glucuronyltransferase 1	0.99 ± 0.21	1.85 ± 0.77	0.018
P06731	Carcinoembryonic antigen-related cell adhesion molecule 5	0.77 ± 0.11	1.52 ± 0.48	0.006
P07711	Cathepsin L1	0.84 ± 0.19	1.57 ± 0.53	0.015
Q05707	Collagen alpha-1(XIV) chain	0.26 ± 0.03	3.56 ± 3.59	0.013
P07358	Complement component C8 beta chain	0.54 ± 0.12	1.75 ± 0.20	0.0005
P01036	Cystatin-S	0.18 ± 0.06	2.91 ± 0.68	0.00004
P09228	Cystatin-SA	0.13 ± 0.14	2.65 ± 0.76	0.008
P01037	Cystatin-SN	0.23 ± 0.10	2.54 ± 0.41	0.0007
P54108	Cysteine-rich secretory protein 3	0.74 ± 0.30	1.78 ± 0.63	0.010
Q02487	Desmocollin-2	0.84 ± 0.25	1.49 ± 0.31	0.023
P32926	Desmoglein-3	0.79 ± 0.25	1.56 ± 0.35	0.018
P27487	Dipeptidyl peptidase 4	0.75 ± 0.22	1.61 ± 0.62	0.026
O94919	Endonuclease domain-containing 1 protein	0.81 ± 0.13	1.61 ± 0.40	0.002
P29317	Ephrin type-A receptor 2	0.84 ± 0.09	1.28 ± 0.33	0.025
Q9UBX5	Fibulin-5	0.73 ± 0.23	1.42 ± 0.50	0.029
P09958	Furin	0.66 ± 0.37	2.03 ± 1.00	0.030
Q9BPY8	Homeodomain-only protein	0.79 ± 0.25	1.76 ± 0.81	0.024
A0A0C4DH69	Immunoglobulin kappa variable 1-9	0.80 ± 0.17	1.54 ± 0.55	0.031
O43278	Kunitz-type protease inhibitor 1	0.88 ± 0.07	1.18 ± 0.24	0.025
P22079	Lactoperoxidase	0.87 ± 0.10	1.51 ± 0.62	0.032
P31025	Lipocalin-1	0.25 ± 0.13	2.09 ± 1.39	0.005
P40121	Macrophage-capping protein	0.77 ± 0.18	1.45 ± 0.19	0.010
P08571	Monocyte differentiation antigen CD14	0.78 ± 0.20	1.53 ± 0.50	0.014
Q96NY8	Nectin-4	0.94 ± 0.19	1.30 ± 0.23	0.037
P19021	Peptidyl-glycine alpha-amidating monooxygenase	0.74 ± 0.26	1.75 ± 0.78	0.034
Q9H008	Phospholysine phosphohistidine inorganic pyrophosphate phosphatase	0.69 ± 0.11	0.90 ± 0.08	0.031
P12273	Prolactin-inducible protein	0.59 ± 0.31	2.28 ± 0.81	0.007
Q16378	Proline-rich protein 4	0.44 ± 0.31	2.82 ± 3.02	0.015
P10586	Receptor-type tyrosine-protein phosphatase F	0.97 ± 0.21	1.56 ± 0.47	0.033
Q8WVQ1	Soluble calcium-activated nucleotidase 1	0.89 ± 0.09	1.33 ± 0.31	0.022
P07996	Thrombospondin-1	0.79 ± 0.11	1.15 ± 0.28	0.039
Q9UL52	Transmembrane protease serine 11E	0.83 ± 0.16	1.38 ± 0.34	0.013
P25311	Zinc-alpha-2-glycoprotein	0.89 ± 0.15	1.58 ± 0.43	0.008
Higher in cancer patients				
O00232	26S proteasome non-ATPase regulatory subunit 12	1.04 ± 015	0.83 ± 0.11	0.048
P10155	60 kDa SS-A/Ro ribonucleoprotein	1.43 ± 0.26	0.68 ± 0.21	0.002
P39687	Acidic leucine-rich nuclear phosphoprotein 32 family member A	1.42 ± 0.61	0.60 ± 0.37	0.044
Q92688	Acidic leucine-rich nuclear phosphoprotein 32 family member B	1.15 ± 0.40	0.57 ± 0.15	0.025
P27797	Calreticulin	1.40 ± 0.78	0.49 ± 0.30	0.043
Q07021	Complement component 1 Q subcomponent-binding protein, Mitochondrial	1.48 ± 0.28	0.90 ± 0.27	0.017
P00751	Complement factor B	0.99 ± 0.13	0.72 ± 0.12	0.013
Q12882	Dihydropyrimidine dehydrogenase [NADP(+)]	1.25 ± 0.18	0.93 ± 0.20	0.032
P24534	Elongation factor 1-beta	1.05 ± 0.15	0.65 ± 0.18	0.015
P11021	Endoplasmic reticulum chaperone BiP	1.11 ± 0.06	0.78 ± 0.16	0.013
P00367	Glutamate dehydrogenase 1, mitochondrial	1.15 ± 0.11	0.78 ± 0.22	0.025
P09488	Glutathione S-transferase Mu 1	1.62 ± 1.09	0.13 ± 0.02	0.006
P07900	Heat shock protein HSP 90-alpha	1.18 ± 0.14	0.55 ± 0.23	0.010
P08238	Heat shock protein HSP 90-beta	1.13 ± 0.27	0.53 ± 0.23	0.025
P0DOX5	Immunoglobulin gamma-1 heavy chain	1.04 ± 0.35	0.59 ± 0.21	0.037
Q12906	Interleukin enhancer-binding factor 3	1.16 ± 0.19	0.74 ± 0.22	0.027
Q7Z4W1	L-xylulose reductase	1.11 ± 0.12	0.66 ± 0.23	0.017
P09237	Matrilysin	1.17 ± 0.26	0.61 ± 0.20	0.009
P29966	Myristoylated alanine-rich C-kinase substrate	0.99 ± 0.08	0.72 ± 0.12	0.008
Q6P4A8	Phospholipase B-like 1	1.04 ± 0.45	0.48 ± 0.21	0.038
Q14435	Polypeptide N-acetylgalactosaminyltransferase 3	1.05 ± 0.11	1.35 ± 0.17	0.016
P13667	Protein disulfide-isomerase A4	1.22 ± 0.34	0.46 ± 0.31	0.011
Q15084	Protein disulfide-isomerase A6	1.00 ± 0.04	0.74 ± 0.15	0.028
Q15435	Protein phosphatase 1 regulatory subunit 7	1.04 ± 0.10	0.71 ± 0.26	0.043
P31949	Protein S100-A11	1.06 ± 0.12	0.79 ± 0.14	0.015
Q01105	Protein SET	1.46 ± 0.94	0.53 ± 0.20	0.043
P06454	Prothymosin alpha	1.16 ± 0.58	0.29 ± 0.27	0.013
Q9NQC3	Reticulon-4	1.21 ± 0.24	0.73 ± 0.26	0.042
P50453	Serpin B9	1.16 ± 0.25	0.68 ± 0.24	0.028
P35542	Serum amyloid A-4 protein	0.93 ± 0.18	0.50 ± 0.18	0.012
P02743	Serum amyloid P-component	0.98 ± 0.32	0.53 ± 0.18	0.043
Q9Y6N5	Sulfide:quinone oxidoreductase, mitochondrial	1.49 ± 0.21	0.76 ± 0.25	0.007
P04179	Superoxide dismutase [Mn], mitochondrial	1.26 ± 0.50	0.63 ± 0.19	0.042
P61586	Transforming protein RhoA	1.09 ± 0.23	0.75 ± 0.19	0.042

The relative abundances of 5 salivary gland proteins were significantly lower in the cancer group 6 months post treatment compared with the control group: Cystatin S, SA, SN, Cystein-rich secretory protein 3, Lactoperoxidase (Fig. 1a), Prolactin-inducible protein and Proline-rich protein 4 (Fig. 1b). There were tendencies to lower abundances of basic salivary proline-rich protein 2 (p = 0.06), Cystatin D (p = 0.06), and Mucin MUC7 (p = 0.07).

### 3.3 Proteins in cancer patients at pre-treatment compared with 6 months post-treatment

Eleven of the proteins were found in lower relative abundances at pre-treatment compared with 6 months post-treatment (5 cell-associated, 3 enzymes, 2 associated with the immune system, 1 salivary gland protein), and 15 proteins in higher (6 cell-associated, 3 enzymes, 3 salivary gland proteins, 2 associated with the immune system, 1 other) (Table 4).

**Table 4:** Proteins showing statistically significant differences in relative abundances (%)for the cancer patients pre-treatment compared with 6 months post-treatment.

Accession	Protein name	Cancer	6 mo post	p-value
Number		pre-treatment	treatment	
Lower pre-treatment compared with 6 months post-treatment				
P10155	60 kDa SS-A/Ro ribonucleoprotein	0.92 ± 0.17	1.43 ± 0.26	0.008
P04083	Annexin A1	0.69 ± 0.21	1.21 ± 0.42	0.040
P13688	Carcinoembryonic antigen-related cell adhesion molecule 1	0.68 ± 0.23	1.05 ± 0.16	0.045
P13987	CD59 glycoprotein	0.68 ± 0.16	1.07 ± 0.30	0.033
Q01469	Fatty acid-binding protein 5	0.68 ± 0.15	1.20 ± 0.38	0.033
P00367	Glutamate dehydrogenase 1, mitochondrial	0.92 ± 0.14	1.15 ± 0.11	0.026
P40926	Malate dehydrogenase, mitochondrial	0.89 ± 0.05	1.11 ± 0.14	0.035
O95969	Secretoglobin family 1D member 2	0.54 ± 0.27	1.33 ± 0.72	0.026
Q9Y6N5	Sulfide:quinone oxidoreductase, mitochondrial	0.84 ± 0.11	1.49 ± 0.21	0.001
P20061	Transcobalamin-1	0.84 ± 0.20	1.10 ± 0.07	0.049
Q92890	Ubiquitin recognition factor in ER-associated degradation protein 1	0.83 ± 0.14	1.14 ± 0.20	0.043
Higher pre-treatment compared with 6 months post-treatment				
O43598	2′-deoxynucleoside 5′-phosphate N-hydrolase 1	1.23 ± 0.18	0.77 ± 0.26	0.036
P55008	Allograft inflammatory factor 1	1.55 ± 0.61	0.86 ± 0.30	0.036
Q9GZN4	Brain-specific serine protease 4	1.16 ± 0.18	0.92 ± 0.03	0.030
Q96CX2	BTB/POZ domain-containing protein KCTD12	1.36 ± 0.58	0.74 ± 0.15	0.042
Q05707	Collagen alpha-1(XIV) chain	0.66 ± 0.29	0.26 ± 0.03	0.009
P07358	Complement component C8 beta chain	1.08 ± 0.31	0.54 ± 0.12	0.004
P01036	Cystatin-S	1.41 ± 1.04	0.18 ± 0.06	0.002
P09228	Cystatin-SA	1.44 ± 1.40	0.13 ± 0.14	0.031
P01037	Cystatin-SN	1.29 ± 0.93	0.23 ± 0.10	0.003
P23588	Eukaryotic translation initiation factor 4B	1.34 ± 0.40	0.67 ± 0.17	0.013
P31025	Lipocalin-1	1.17 ± 1.05	0.25 ± 0.13	0.026
O60664	Perilipin-3	1.25 ± 0.27	0.84 ± 0.17	0.026
O60610	Protein diaphanous homolog 1	1.32 ± 0.34	0.89 ± 0.17	0.030
P22061	Protein-L-isoaspartate(D-aspartate) O-methyltransferase	1.46 ± 0.64	0.81 ± 0.19	0.032
P50991	T-complex protein 1 subunit delta	1.19 ± 0.22	0.90 ± 0.14	0.047

### 3.4 Abundances of mucins and problems with dry mouth, sticky saliva and swallowing

**Fig. 2:**
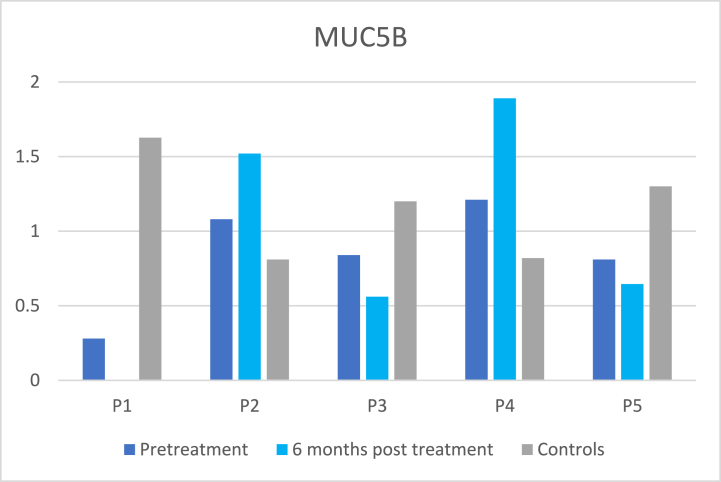

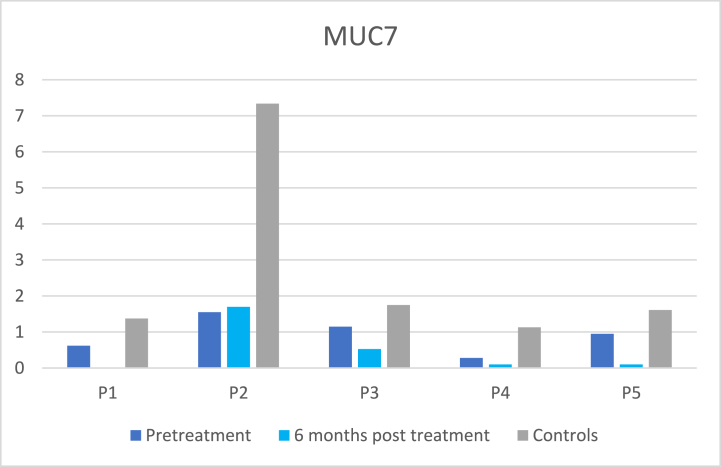
Fig. 2a.Relative abundances of MUC5B in the cancer patients at pre-treatment and at 6 months post-treatment and in their matched control. Mean ± SD relative abundances were: cancer pretreatment 0.85 ± 0.36, cancer 6 months post-treatment 1.15 ± 0.66, healthy controls 1.15 ± 0.35. Value for P1 at post-treatment is missing. Fig. 2b Relative abundances of MUC7 in the cancer patients at pre-treatment and at 6 months post-treatment and in their matched control. Mean ± SD relative abundances were: cancer pretreatment 0.91 ± 0.49, cancer 6 months post-treatment 0.61 ± 0.75, healthy controls 2.64 ± 2.64. Value for P1 at post-treatment is missing.

As can be seen in Fig. 2a and b, there were large differences between relative abundances of the mucins MUC5B and MUC7 and patients’ problems with dry mouth, sticky saliva (Table 5), and swallowing and there were no statistically significant correlations found at six months post-treatment.

**Table 5:** Patient-reported problems with dry mouth, sticky saliva and swallowing obtained from the EORTC HN35 questionnaire. 1 = not at all, 2 = a little, 3 = quite a bit and 4 = very much. For swallowing a median value for 4 questions: problems swallowing liquids, problems swallowing pureed foods, problems swallowing solid foods, and if they had choked when swallowing is shown.

Patient	Dry mouth		Sticky saliva		Problems swallowing	
	Pretreat-ment	6 months post treatment	Pretreat-ment	6 months post treatment	Pretreat-ment	6 months post treatment
P1	2	4	1	2	4	1
P2	1	3	1	1	1	2
P3	4	4	4	4	1	2
P4	1	2	1	2	1	1
P5	1	2	1	3	2	4

## 4 Discussion

In the present explorative study, the proteome in stimulated whole saliva collected from patients with cancer of the head and neck region at pre-treatment and 6 months post-treatment was analyzed and compared with that of healthy controls matched according to age and gender. The cancer patients had lower relative abundances of five proteins produced by the salivary glands post-treatment, which might lead to a reduced defense against oral disorders. No clear relation between mucins and dry mouth, sticky saliva, and problems with swallowing was detected.

### 4.1 Cystatins

The relative abundance of Cystatin-SN was significantly lower compared with healthy subjects at pre-treatment, and six months post-treatment and there were lower relative abundances of Cystatin S, Cystatin SA, and Cystatin SN. Cystatins are inhibitors of cysteine proteinases and have antiviral and antibacterial functions. Cystatins could play a protective and regulatory role under inflammatory conditions. The submandibular glands produce the largest amount of Cystatin S followed by palatine minor glands, while the sublingual glands produce a lower amount of Cystatin S and the parotid gland almost no Cystatin S [20]. Cystatin S has been found to bind more calcium and bind more rapidly to calcium apatite compared to Cystatin SA or SN [21]. The results of the present study are in accordance with previous studies reporting decreased abundances of Cystatin S, Cystatin-SA, and Cystatin-SN in unstimulated saliva of head and neck cancer patients 3–4 months post-treatment [18] and decreased levels of Cystatin D, S, SA, and SN in stimulated saliva at ≥ 6 months post-treatment [22]. In contrast, in stimulated parotid saliva ≥6 months post-treatment of head and neck cancer increased concentrations of cystatins were found compared with healthy controls [23]. The marked decrease of Cystatins at post-treatment might lead to a lower ability of saliva to bind calcium, which as a result may reduce the remineralization capacity of tooth surfaces and could contribute to dry mouth problems.

### 4.2 Lactoperoxidase

In the present study, the cancer patients had a lower relative abundance of Lactoperoxidase at 6 months post-treatment compared with the healthy controls. The enzyme Lactoperoxidase is a calcium- and iron-containing glycoprotein [24] secreted by epithelial cells of the acinus in submandibular and parotid salivary glands [25]. Lactoperoxidase can be adsorbed onto the salivary pellicle and can prevent the adhesion of cariogenic microorganisms [25]. In accordance with the literature, our study supports that the lower abundance of Lactoperoxidase might contribute to deteriorated defense against microorganisms associated with caries, periodontal disease, and fungal infections [25].

### 4.3 Cysteine-rich secretory protein 3

In the present study, there was a lower abundance of Cysteine-rich secretory protein 3 (CRISP3) in the cancer patients at 6 months post-treatment compared with healthy controls. CRISP3 is expressed by human labial glands [26], the sublingual glands, and to a lesser extent by the submandibular glands, while the parotid glands do not express CRISP3 [27]. The role of CRISP3 in saliva is unknown. Other studies have not found any changes in CRISP3 in stimulated saliva from cancer patients at ≥ 6 months post-treatment [18,22] which might be due to differences in sensitivity in methods used since CRISP3 is a small protein, 28 kDa.

### 4.4 Proline-rich proteins

Proline-rich protein 4 was found in lower relative abundance in the cancer patients 6 months post-treatment compared with the healthy controls. Proline-rich proteins (PRPs) constitute 20–25 % of all proteins in saliva and are the largest group of proteins secreted by parotid glands but are also produced by the submandibular glands [28]. A decrease in PRP 4 was reported in unstimulated saliva in cancer patients pre-treatment compared with healthy controls by Ventura et al. [18], while an increase of PRPs in stimulated parotid saliva was found ≥6 months post cancer treatment [23]. Plausible explanations are differences in saliva used; whole saliva as in the present study and in the study by Ventura et al. [18], or directly from the parotid glands [23], treatment of saliva before analysis; uncentrifuged as in the present study and in the study by Laheij et al. [23], or centrifuged at 4500×*g* for 10 min [18]. The methods used for proteome analysis also differed.

### 4.5 Mucins

The mucins MUC5B and MUC7 are the most abundant mucins in the oral cavity. MUC5B and MUC7 interact with oral microbes to facilitate their removal and/or reduce their pathogenicity [29]. Mucins are also important for the lubrication of the oral mucosal membranes. The relative abundance of Mucin MUC7 tended to be lower in the cancer patients both at pre-treatment and at 6 months post-treatment compared with healthy controls, which is in accordance with previous studies [15,16]. No significant difference in the abundance of MUC5B was detected in the present study, which is in accordance with the results of our previous study analysing MUC5B in cancer patients 6 months post-treatment compared with healthy controls using an ELISA-method [30]. Dijkema et al. [28], reported lower levels of MUC5B in submandibular saliva at 12 months post-treatment compared with pre-treatment. In the present study, the patients with secretion rates of 0.8 ml/min and 0.5 ml/min both had a lower abundance of MUC5B and reported “a little” problems with dry mouth.

Problems with swallowing, senses, dry mouth and sticky saliva has been found to be increased at 6 months post cancer treatment compared with baseline [31]. We found that problems with insomnia, swallowing, social eating, dry mouth, and sticky saliva were especially pronounced in those who had hyposalivation (≤0.7 ml/min) compared with those with higher secretion rates at 6 months post-treatment compared with baseline [6]. The results of the present study indicate that both the reduction in salivary secretion rate and a reduced level of mucin MUC7 might be of importance for patients' experience of dry mouth and sticky saliva and swallowing difficulties. More studies including a higher number of patients are needed to further explore how saliva composition and secretion rate affect patients’ experience.

### 4.6 Methodological considerations

In the present study, stimulated whole saliva which had been stored at −80 °C and thawed on ice was used for the analysis of the proteome. Whole saliva is a mixture of water (99 %), proteins, electrolytes, bacteria and epithelial cells, blood cells (neutrophils) and other tissue fluid proteins from the gingival crevicular fluid [32]. The proteins vary a lot in size from a few Daltons (Da) to 100–1000 MDa. Also, proteins build networks including several different proteins [33]. Centrifugation of samples is often used to remove epithelial cells and debris, but centrifugation is likely to also remove proteins and networks of proteins such as mucins [34]. The drawback of freezing saliva without centrifugation is that epithelial cells and neutrophils will burst when thawing the saliva leading to the release of intracellular proteins. There are variations regarding differences in proteins detected in different studies, which most likely is due to the collection of saliva, methods for preparing the saliva for analysis, and how the samples are analyzed. A suggestion for future studies on the salivary proteome is to analyze saliva with and without centrifugation prior to freezing the samples and the analyze both stimulated and unstimulated saliva.

### 4.7 Study limitations

There was a low number of cancer patients included in the present explorative study. Therefore, further studies including more patients are needed to increase the knowledge about changes in the saliva proteome and especially regarding proteins secreted from the salivary glands. To confirm the MS results by analysis of key proteins being statistically significant between cancer patients and controls between pretreatment and six months post cancer treatment using enzyme-linked immunosorbent assay or similar methods would also have strengthen the results of the study.

### 4.8 Clinical relevance

An increased knowledge about the saliva proteome and correlations with patient's problems due to reduced salivary secretion rate is important both for giving better advice to relieve dry mouth problems and for the development of more effective products.

## 5 Conclusions

The most striking differences in stimulated saliva proteome between the cancer patients and healthy controls in this explorative study were the decreased relative abundances of cystatins both pre-treatment and 6 months post-treatment as well as reduced relative abundances of 4 other saliva proteins post-treatment. The relative abundance of mucin MUC7 tended to be decreased in the cancer patients compared with the healthy controls, which might be of importance for patients’ experience of dry mouth, sticky saliva and swallowing difficulties.

## CRediT authorship contribution statement

**Ulrica Almhöjd:** Writing – review & editing, Writing – original draft, Methodology, Formal analysis, Conceptualization. **Amela Fisic:** Writing – review & editing, Writing – original draft, Formal analysis, Conceptualization. **Hülya Cevik-Aras:** Writing – review & editing, Writing – original draft, Resources, Formal analysis, Conceptualization. **Lisa Tuomi:** Writing – review & editing, Writing – original draft, Resources. **Caterina Finizia:** Writing – review & editing, Writing – original draft, Resources. **Annica Almståhl:** Writing – review & editing, Writing – original draft, Project administration, Investigation, Funding acquisition, Formal analysis, Data curation, Conceptualization.

## Ethics approval and consent to participate

This study was reviewed and approved by the Swedish Ethical Review Authority, with the approval numbers: [Dnr 1151-18 and Dnr 2019-00752 for the cancer patients and Dnr 2020–03094 for the healthy controls]. All participants provided informed consent to participate in in the study and for the publication of their anonymized case details. All methods were carried out in accordance with relevant guidelines and regulations.

## Consent for publication

Not applicable.

## Data availability statement

The mass spectrometry proteomics data have been deposited to the ProteomeXChange Consortium via the PRIDE partner repository with the dataset identifier PXD047500.

## Funding

This study was supported by grants from TUA Research Funding; The 10.13039/501100005761Sahlgrenska Academy at 10.13039/501100005760University of Gothenburg/Region Västra Götaland, Sweden, 10.13039/501100002794Swedish Cancer Society and FRF-foundation, Assar Gabrielsson Foundation and the Sjöberg Foundation.

## Declaration of competing interest

The authors declare that they have no known competing financial interests or personal relationships that could have appeared to influence the work reported in this paper.
